# Mpox (Monkeypox) and the Eye: Ocular Manifestation, Diagnosis, Treatment and Vaccination

**DOI:** 10.3390/v15030616

**Published:** 2023-02-23

**Authors:** Yuan Zong, Koju Kamoi, Jing Zhang, Mingming Yang, Kyoko Ohno-Matsui

**Affiliations:** Department of Ophthalmology and Visual Science, Graduate School of Medical and Dental Sciences, Tokyo Medical and Dental University, Tokyo 113-8510, Japan

**Keywords:** mpox, monkeypox, eye disease, virus, literature review

## Abstract

At present, the world is at the tipping point of the outbreak of mpox. The World Health Organization has declared the current mpox outbreak a ‘public health emergency of international concern’. Mpox has been shown to be associated with several ocular manifestations. Given the current state of the mpox outbreak, healthcare providers, particularly ophthalmologists, need to be aware of these ophthalmic symptoms and how to manage them. In this review, we highlight current knowledge on the ocular symptoms of mpox virus (MPXV) infections and how to detect them. In addition, we summarize the treatment strategies for these ocular manifestations of MPXV infections and outline the relationship between vaccination and the ocular symptoms of mpox.

## 1. Introduction

Mpox (previously called monkeypox) is a viral zoonosis, and in humans, it shares several clinical characteristics with smallpox [1,2]. Although smallpox has been eradicated worldwide since 1980, mpox is still endemic in parts of Central and Western Africa [3,4,5]. However, it should be noted that most of the mpox cases recorded in the 2022 outbreak occurred in Western and European countries, with no apparent epidemiological link between the cases in these regions [3]. The number of mpox cases has been increasing daily since early May 2022, when the first cases of 2022 were reported in the United Kingdom (UK), Spain, and other parts of Europe [6,7]. Unlike previous mpox outbreaks, most cases in this outbreak have been reported from the European region and the Americas, with young men being particularly affected by the outbreak and the majority being gay, bisexual, or other men who have had sex with men [8]. The World Health Organization (WHO) has designated the current mpox outbreak a ‘public health emergency of international concern’ [9]. Additionally, the Department of Health and Human Services declared a public health emergency on 4 August 2022, in response to the mpox outbreak in the United States (US), which began on 17 May 2022 [10].

It has been demonstrated that mpox is associated with several ocular manifestations [11]. Considering the current state of the mpox outbreak, healthcare providers, especially ophthalmologists and optometrists, need to be aware of these ocular manifestations. In this review, we highlight current knowledge on the ocular manifestations of mpox and summarize their treatment strategies. In addition, we outline the relationship between vaccination and the ocular symptoms of mpox.

## 2. General Characteristics of the Mpox Virus

The mpox virus (MPXV) is a double-stranded DNA virus that belongs to the orthopoxvirus genus. Aside from MPXV, the other three orthopox viruses that are pathogenic to humans are the large variant virus that causes smallpox, which has been eradicated; the small variant virus; and the cowpox virus [12,13]. The natural host of MPXV is unknown; however, rodents, rabbits, bats, and non-human primates can contract the virus [14,15]. MPXV is classified into two distinct genetic clades, historically identified according to the geographical association but renamed by the WHO in an effort to reduce discrimination and stigma [16]. In the past, Clade I (formerly known as the Congo Basin clade) was responsible for several serious outbreaks of disease throughout history [17,18]. In contrast, Clade II (formerly the West African clade) is associated with the current global mpox outbreak in 2022, which is dominated by Europe and North America [19]. The findings of several serological studies suggest that MPXV is primarily maintained by mammalian species in Africa, which may explain why MPXV was only prevalent in Africa in the past [12,20,21,22]. Humans may also become infected with MPXV from direct or indirect contact with the skin lesions, body fluids, or respiratory droplets of infected animals, which facilitate human-to-human transmission [7,23,24,25]. In addition, vertical transmission of mpox in humans has been observed in stillborn babies born in the second trimester to mothers infected with mpox [26,27].

MPXV was first isolated from a colony of cynomolgus monkeys in Copenhagen, Denmark, in 1958 [28]. The first case of human mpox was discovered in 1970 in the Basankusu Territory, Democratic Republic of the Congo (DRC). From then on, MPXV infections were occasionally reported in 11 African countries in the following decades [6,21,29,30]. In May 2022, MPXV infections re-emerged in several countries; however, no clear epidemiological link between the reported cases has been identified. The current outbreak is rapidly expanding throughout Europe and the Western Hemisphere. A total of 75,141 laboratory-confirmed cases and 3453 probable cases, including 31 deaths, have been reported to the WHO as of 19 October 2022, at 17 h CEST [31].

Although mpox has not been shown to be transmitted sexually, the WHO states that close contact is a likely source of transmission [32]. Notably, human-to-human transmission, especially between men who have sex with men (MSM), appears to account for most of the cases recorded during the current global mpox outbreak [11,25,27,33]. Thus, patients with mpox during this outbreak tend to present first to clinics because of anogenital lesions. These patients tend to be treated quickly by healthcare staff in the well-established healthcare systems of developed countries, which may explain the low proportion of mpox cases with ocular involvement reported in the 2022 mpox outbreak [7,23,24,25].

## 3. Ophthalmic Manifestations of Mpox

The clinical presentation of mpox is similar to that of smallpox. Mpox has an incubation period of five to twenty-one days, is generally mild and self-limiting, and has few serious manifestations or complications. The main systemic symptoms of mpox include fever, intense headache, back pain, lymphadenopathy, myalgia, intense shortness of breath, and rashes; the hallmark symptom of mpox is a disseminated, blistering, pustular rash, which occurs in approximately 90% of cases [3,34,35]. Unlike smallpox or chickenpox, enlargement of the lymph nodes, including those in the upper jaw, neck, and groin, occurs in the early stages of mpox, coinciding with the onset of fever [3,27,36]. In addition, although previous reported mpox cases were mostly characterized by generalized rashes, with the face, trunk, and extremities being heavily affected [37,38], the cases reported in the current 2022 mpox outbreak are mostly characterized by multiple rashes confined to one or two anatomical sites, predominantly the perianal or penile regions [39,40].

**Figure 1:**
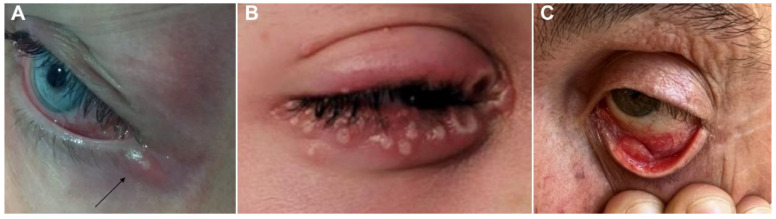
Typical eyelid and conjunctival involvement in mpox. (**A**) Vesicles on the left lower eyelid (black arrow). (**B**) Multiple papular lesions on the right eyelid. (**C**) Ulceration of the palpebral conjunctiva. ((**A**) Adapted with permission from Ref. [42]. 2022, Benatti et al.; (**B**) Adapted with permission from Ref. [47]. 2022, Mazzotta et al.; (**C**) Adapted with permission from Ref. [50]. 2022, De Sousa et al.).

The ophthalmic symptoms of mpox are summarized in Table 1. The most common ocular symptom of MPXV infection is its characteristic rash in the periorbital and orbital areas [11,41]. In a retrospective study conducted by Ogoina et al. between September 2017 and December 2018, 25% of 40 patients with mpox admitted to hospitals in Nigeria had rashes in the ocular region [24]. Eyelid and conjunctival involvement are common ocular clinical manifestations of MPXV infection (Figure 1) [11,42]. Conjunctivitis due to MPXV infection may manifest as conjunctival ulcers, disseminated blistering or papular conjunctival lesions, conjunctival follicular reactions, and pseudomembranous/subconjunctival nodules [17]. In a study conducted in the DRC by Hughes et al., 23.1% of patients with mpox in the Tshuapa health district had conjunctivitis [43]. In addition, the patients with mpox who had conjunctivitis reported more frequent symptoms, such as nausea, chills and sweats, mouth ulcers, sore throats, fatigue, lymphadenopathy, and photophobia, than those without conjunctivitis.

Furthermore, the report indicated that conjunctivitis appears to predict the course of mpox, and that immunocompromised people, such as children, tend to be more susceptible to conjunctivitis. The authors also reported that 47% of cases with keratitis reported being bedridden, compared to 16% of patients without keratitis. In the same study, 61.8% of the patients who presented with conjunctivitis were children younger than ten. In another study conducted in Zaire, Janseghers et al. reported a case of an unvaccinated 2.5-year-old child with a confirmed diagnosis of mpox, whose most prominent symptoms were blisters and pustules all over the body, along with bilateral conjunctivitis and palpebral lesions. Despite the appropriate supportive treatment administered in the hospital, the child died ten days later [44]. There have been several reports of conjunctivitis in patients with MPXV infection during the current mpox outbreak [42,45,46]. Benatti et al. reported that a biracial white male in his mid-40s who tested positive for mpox DNA (polymerase chain reaction [PCR]) presented with back muscle pain, fever, and a dry cough that lasted for 48 h, seven days before the blisters appeared. He then developed a small vesicle on his lower eyelid and conjunctivitis in his left eye four days later (Figure 1A) [42]. Mazzotta et al. reported that a 26-year-old Italian male who presented to an outpatient clinic with two papular lesions in the suprapubic region was diagnosed with MPXV infection after real-time PCR testing of swabs from the lesions [47]. He was then hospitalized because he had developed a headache, high temperature, general malaise, swollen inguinal lymph nodes, and several papular lesions on the right eyelid that progressively involved the conjunctiva and periorbital area (Figure 1B). MPXV was detected in swabs of his periorbital lesions and conjunctiva. Notably, the patient presented with systemic symptoms and ocular involvement at similar times, suggesting that ocular localization of MPXV may not be through conjunctival transmission in the early viremia phase of the infection but through self-inoculation. It is also worth noting that in some cases of conjunctivitis in patients with mpox, the final diagnosis was bacterial conjunctivitis [48,49].

**Figure 2:**
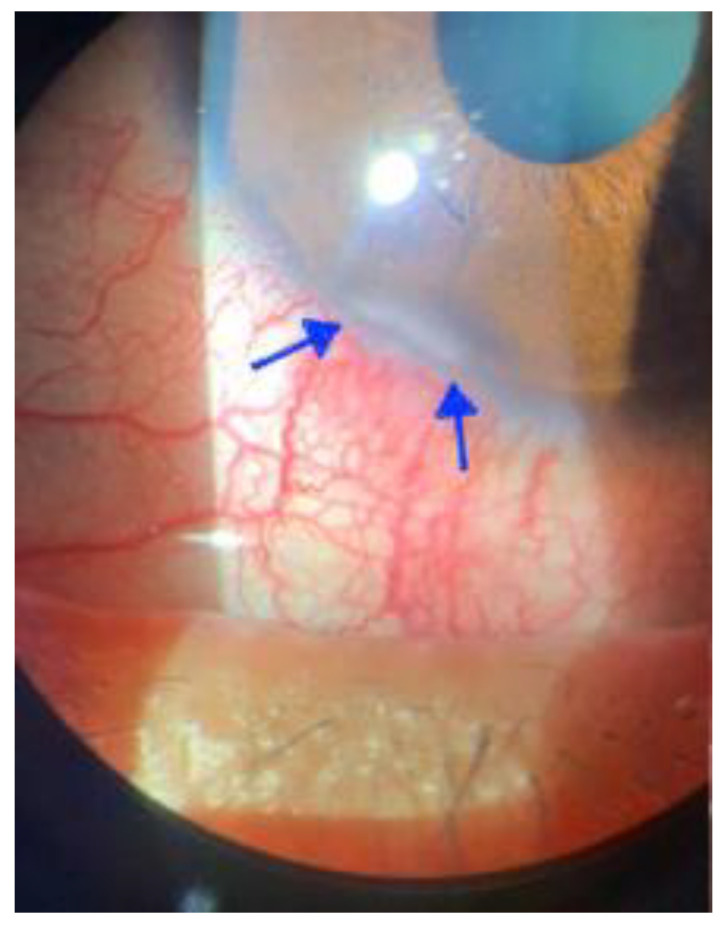
Patient with mpox and HIV infection who presented with peripheral keratitis (blue arrow). (Adapted with permission from Ref. [55]. 2022, Ng, F.Y.C et al.).

Corneal infection is the most severe complication of ocular involvement in mpox, resulting in permanent vision loss and corneal scarring [3,41,51,52]. According to previous studies, corneal infections are relatively uncommon complications of MPXV infection [24,53,54]. Ng et al. described a case of a patient with HIV infection and mpox who presented with peripheral keratitis. The patient’s conjunctival swab returned positive after a mpox PCR test (Figure 2) [55]. In a retrospective study of mpox-infectors in Nigeria before the mpox outbreak in 2022, 7.5% (3/40) of the patients developed severe corneal infections that led to keratitis [24]. In another study, clinical and laboratory examinations of 338 patients with mpox were performed in Zaire between 1981 and 1986. The statistics revealed that keratitis and corneal ulceration were present in 3.6% of patients with animal-derived infections and 4.1% of those with human-derived infections, respectively [53]. There is an ongoing discourse on whether the cause of the corneal symptoms in mpox cases is an MPXV infection or a secondary bacterial infection [13,34]. The severity of corneal infections in patients with mpox varies. Corneal pitting, scarring, or ulceration may occur, eventually leading to severe eye damage, corneal opacities, or even permanent blindness [34,52,54]. The current 2022 mpox epidemic has a preponderance of male infections, so there are concerns about whether there is a gender bias in the complications of mpox. A study reviewing 151 female and 6940 male mpox cases in Spain between April and November 2022 found a higher incidence of complications among women than men with mpox but no significant gender difference in the incidence of keratitis [56].

It may also be of interest to eye care professionals that although no reports have yet emerged in mpox patients, occasional reports of retinitis, chorioretinitis, accommodative palsy, optic neuritis, extraocular muscle palsy, suppurative dacryocystitis, retrobulbar haemorrhage with proptosis, ankyloblepharon, and lagophthalmos with exposure keratitis have emerged in past cases of smallpox [57].

**Table 1:** Ophthalmic manifestations of mpox virus.

Ophthalmic Manifestations of Mpox	Number of Patients	References	Country	Study Design
Vesicular rash in the periorbital and orbital areas	10	Ogoina et al., 2020 [24]	Nigeria	Case series
Conjunctivitis	1	Janseghers et al., 1984 [44]	Democratic Republic of the Congo	Case report
	68	Hughes et al., 2014 [43]	Democratic Republic of the Congo	Case series
	1	Meduri et al., 2022 [46]	Switzerland	Case report
	1	Mazzotta et al., 2022 [47]	Italy	Case report
	1	Benatti et al., 2022 [42]	Italy	Case report
	1	Foos et al., 2022 [45]	The USA	Case report
	2	Ci Ng et al., 2023 [55]	Singapore	Case series
Eyelid involvement	1	Janseghers et al., 1984 [44]	Democratic Republic of the Congo	Case report
	1	Benatti et al., 2022 [42]	Italy	Case report
	1	Foos et al., 2022 [45]	The USA	Case report
	1	Mazzotta et al., 2022 [47]	Italy	Case report
Corneal ulcerations	3	Ogoina et al., 2020 [24]	Nigeria	Case series
	1	Ci Ng et al., 2023 [55]	Singapore	Case series

## 4. Detection of the Mpox Virus in Ocular Secretions

The UK Health and Safety Executive defined a laboratory-confirmed MPXV infection as a positive MPXV PCR test result obtained using any anatomical specimen [25,58]. It is possible to test conjunctival swabs and eyelid capsule fluid from patients with mpox using PCR, which allows for precise analysis of small amounts of ocular samples (such as vitreous fluid or aqueous) [48,59,60,61]. Mazzotta et al. reported that they detected MPXV-DNA in an MSM patient’s eyelid and conjunctival swabs with mpox using real-time PCR. Notably, reproducible and infectious MPXV has been successfully isolated in cell culture using conjunctival swabs [47]. This is one of the first descriptions of the isolation of infectious and replication-competent MPXV from the conjunctival swab cultured from a case of mpox with ocular involvement. Meduri et al. reported that in a case of a 39-year-old man who presented with unilateral red eye and pruritus, a slit lamp examination revealed a conjunctival follicular reaction with small white blisters on the nasal bulbar conjunctiva five days after a positive skin PCR for MPXV. During follow-up, the authors noted that the remaining anterior and posterior areas were affected; however, the contralateral eye remained unaffected [46]. Notably, this patient’s viral load in conjunctival and ocular secretions was indirectly similar to that in cutaneous lesions (26.7 versus 24.8 [cycle threshold], respectively). This finding suggests the potential for the transmission of MPXV through ocular contact and highlights the need for healthcare workers to adopt appropriate personal protective measures during ophthalmic examinations of patients with MPXV infections.

## 5. Treatment of Mpox Virus Eye Infections

Most mpox cases are mild and self-limiting. There is no available standard-of-care treatment for MPXV infection to date except for supportive care and symptomatic management [12,39,62]. Nutritional deficiencies and fluid loss can lead to an increased incidence of complications with mpox, which can be effectively mitigated by effective nutritional supplementation and adequate rehydration [54]. For patients with severe symptoms and immunocompromised immune systems, working knowledge of smallpox treatment suggests that vaccines, such as cidofovir, brincidofovir, tecovirimat, and vaccinia immune globulin (VIG), may be effective for the treatment of mpox [35,61,63]. Tecovirimat (Tpoxx) is a low-molecular-weight orthopoxvirus inhibitor developed to treat smallpox. As a result of its specific effectiveness against many orthopox viruses, including those that cause smallpox, cowpox, rabbitpox, and mpox, Tpoxx has been widely used in treating these diseases [3,63,64]. It was licensed for the treatment of mpox by the European Medicines Agency in 2022. Besides, the US Centers for Disease Control and Prevention (CDC) holds expanded access to an investigational new drug protocol for mpox [3,65,66]. In a report of the first patients with MPXV infection in the US treated with Tpoxx, 230 (72.6%) of 317 patients with available outcome information recovered with or without sequelae on or before completion of the post-treatment assessment [67]. Several case reports suggest that Tpoxx is effective for treating human cowpox eye infections [68,69].

Arguments have been made against using steroid eye drops to control ocular inflammation in patients suffering from mpox [34,54]. A patient was reported to have developed recurrent corneal erosions owing to cowpox virus infection nine months after a conjunctival swab returned negative results for the cowpox virus [70]. Using steroid eye drops to control inflammation is suspected to be the cause of this patient’s persistent infection and prolonged corneal damage. The application of lubricants to the eyes and the intake of vitamins have been shown to be effective in preventing corneal infections in patients with smallpox [54]. These measures may also be effective for treating patients with mpox who develop corneal diseases.

## 6. Relationship between Vaccination and Ocular Symptoms of Mpox

There is no licensed, dedicated MPXV vaccine, but smallpox vaccination provides cross-over protection against orthopoxvirus infection. For MPXV infections, historical data suggest protection of approximately 85% [71,72]. Animal studies have also shown that smallpox vaccination confers cross-protection against mpox in monkeys [13,73]. With the eradication of smallpox in the 1980s, smallpox vaccination was discontinued worldwide, making it rare for people under 40 to be vaccinated against smallpox [74]. The eradication of smallpox and the discontinuation of the smallpox vaccine are believed to have created an ecological niche for immunity to mpox [52]. Furthermore, mpox is associated with more serious sequelae and complications in the unvaccinated than smallpox (74% vs 39.5%) [51]. In the field of ophthalmology, vaccines also appear to be effective in preventing mpox eye infections, with one study showing that 7% of patients vaccinated against smallpox developed conjunctivitis and blepharitis, compared with 30% of unvaccinated patients [75]. As described in the CDC guidelines, two vaccines, JYNNEOS (MVA-BN; Imvamune or Imvanex) and ACAM2000, are available to prevent mpox disease [76]. During the current outbreak, JYNNEOS is the primary vaccine used in the United States. It is a third-generation, modified, attenuated Ankara vaccine previously approved for smallpox protection. In addition to the USA, JYNNEOS has also been licensed in Europe and Canada. It is generally accepted that complete immunity can be achieved approximately two weeks after the second dose of JYNNEOS [35,72]. In the UK, immunization with JYNNEOS has been offered off-label as a pre-exposure prophylaxis to those at risk [35,72].

It should be noted that the vaccine strains may cause unintended infections [77]. VIG is used to treat certain complications that occur after vaccination. Additionally, the CDC has an expanded access protocol that allows treating orthopoxvirus, including MPXV, in an outbreak using VIG [78]. For ophthalmologists, it is worth noting that VIG is contraindicated in cases of isolated vaccinia keratitis [35,79]. Over the five years from 1963 to 1968, 336 ocular vaccine infections and 22 corneal vaccine infections were identified through follow-up with patients who received VIG. Most of these patients (70%) were first-time vaccinees [80].

## 7. Nosocomial Infection Prevention and Control

Considering that MPXV can be transmitted through close contact with infected persons, contaminated objects, droplet transmission, and the potential for transmission through ocular secretions, it is necessary for ophthalmologists to learn about the prevention of nosocomial infections of MPXV [50,81]. For people infected with MPXV, regular hand cleaning and disinfection are required to avoid ocular complications. Meanwhile, contact with the eyes in the presence of active skin lesions should be avoided. Contact lens use is not recommended at any stage of the infection [72]. For ophthalmologists, in addition to self-protection with appropriate personal protective equipment (including respiratory protection) when interacting with confirmed or suspected cases of mpox, reusable ophthalmic equipment, including slit lamps, ophthalmic lenses, and indirect inspection glasses, must also be decontaminated in strict compliance with local infection control guidelines [82]. Prior vaccination is highly recommended for healthcare workers and hospital staff involved in clinical testing who are at risk of exposure to MPXV [35].

## 8. Conclusions

Information regarding ocular involvement during the current mpox outbreak remains quite limited and fragmented. However, given the continued rapid increase in mpox cases and the proportion of cases with ocular involvement in previously endemic areas, ophthalmologists should consider mpox as a differential diagnosis duration examination. In addition, considering the potential for the transmission of MPXV through contact with ocular secretions, healthcare workers need to adopt appropriate personal protective measures when performing eye examinations on patients with MPXV infection.

## Data Availability

All data related to this study are presented and published here.
